# Raman Spectra and Excitonic Effects of the Novel Ta_2_Ni_3_Te_5_ Monolayer

**DOI:** 10.1021/acsomega.4c09075

**Published:** 2024-11-28

**Authors:** Alexandre C. Dias, Raphael M. Tromer, Humberto R. Gutiérrez, Douglas S. Galvão, Elie A. Moujaes

**Affiliations:** † Institute of Physics and International Center of Physics, University of Brasília, Brasília 70919-970, DF, Brazil; ‡ State University of Campinas and Center for Computational Engineering and Sciences, Campinas 13083−970, SP,Brazil; § Department of Physics, University of South Florida, Tampa, Florida 33620, United States; ∥ Physics Department, Federal University of Rondônia, 76801−974 Porto Velho, Brazil

## Abstract

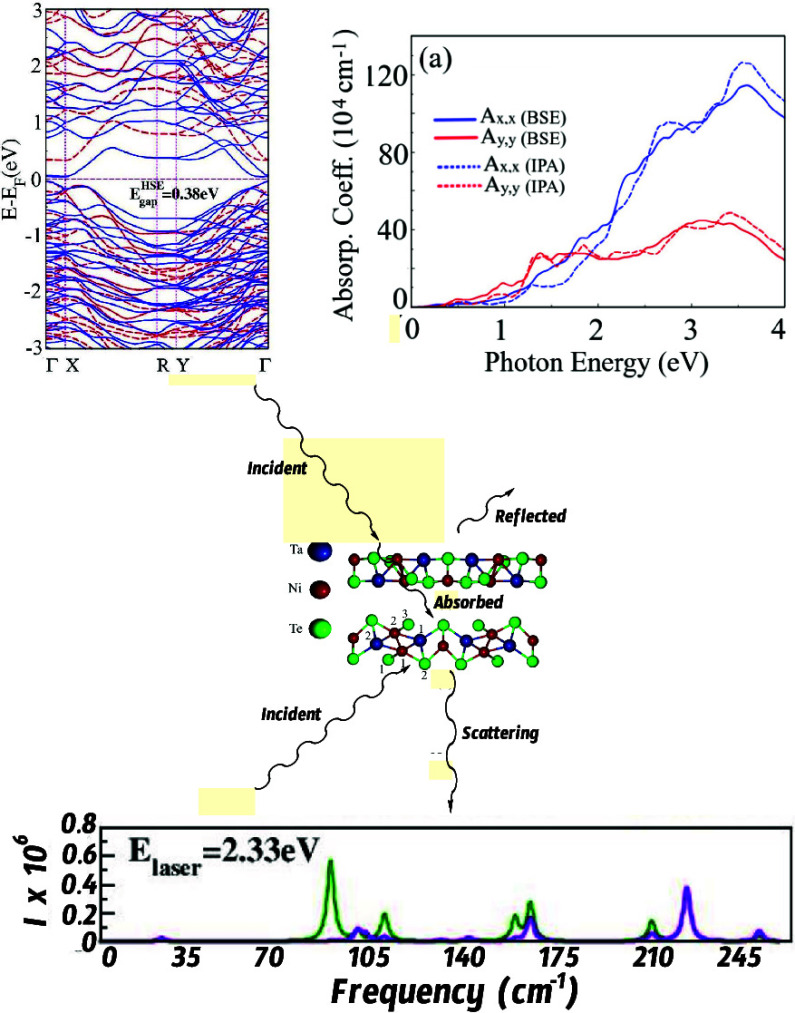

We have investigated the Raman spectrum and excitonic effects of the novel 2D Ta_2_Ni_3_Te_5_ structure. The monolayer is an indirect band gap semiconductor with an electronic band gap value of 0.09 and 0.38 eV, determined using GGA-PBE and HSE06 exchange-correlation functionals, respectively. Since this structure is energetically, dynamically, and mechanically stable, it could be synthesized as a free-standing material. We identify 10 Raman- and 10 infrared-active modes for various laser energies, including those commonly used in Raman spectroscopy experiments. It was also observed that the contribution of Ni atoms is minimal in most Raman vibrational modes. In contrast, most infrared vibrational modes do not involve the vibration of the Ta atoms. As far as the optical properties are concerned, this monolayer shows a robust linear anisotropy, an exciton binding energy of 287 meV, and a high reflectivity in the ultraviolet region, which is more intense for linear light polarization along the *x* direction.

## 1 Introduction

Graphene’s experimental realization in the early 2000s^1,2^ marked a new frontier in solid-state physics and materials science. Graphene exhibits unique electronic and mechanical properties.^3^ Graphene also renewed the interest in other new 2D materials, some of which possess interesting properties^4−7^ and functionalities,.^8−10^ Examples include the combination of different monolayers to form van der Waals (vdW) heterojunctions, allowing us to tune a wide variety of properties.^11−13^ Unlike some bulk systems, the quantum confinement in those materials makes the excitonic quasi-particle effects relevant for a reliable characterization of their linear optical response,^9,14^ requiring sophisticated experimental and theoretical approaches.

Recently, vdW layered materials of the A_2_M_1,3_X_5_ (A = Ta, Nb; M = Pd, Ni and X = Se, Te) family have received attention due to exotic properties, such as the quantum spin Hall effect in the Ta_2_Pd_3_Te_5_ monolayer,^15,16^ excitons in Ta_2_NiSe_5_,^17,18^ and superconductivity in Nb_2_Pd_3_Te_5_ and doped Ta_2_Pd_3_Te_5_ structures.^19^ Both Ta_2_Pd_3_Te_5_ and Ta_2_Ni_3_Te_5_, which have been experimentally created in their layered bulk form,^20,21^ exhibit intriguing topological properties.^20,22^

Ta_2_Pd_3_Te_5_ and Ta_2_Ni_3_Te_5_ share the same crystal structure,^23^ consisting of rhombus-like Ta_2_Pd_2_(Ta_2_Ni_2_) clusters that act as building blocks within each layer. These clusters are linked into 1D chains and then loosely connected to form a 2D layer.^24^ Despite the structural similarities, both compounds exhibit distinct properties due to differences in the band gap of their normal states.^23^ Ta_2_Pd_3_Te_5_ is classified as an excitonic insulator,^23,25^ whereas Ta_2_Ni_3_Te_5_ is a small band gap semiconductor, with an electronic band gap of 31 meV in its bulk form.^20,23^

In particular, Ta_2_Ni_3_Te_5_ exhibits topological phase transitions under pressure and is also predicted to undergo a similar transition under strain,^15,26^ showing potential for various practical applications. In addition, Ta_2_Ni_3_Te_5_ also presents an in-plane anisotropy, which originates from its anisotropic crystal lattice and point group symmetry;^21^ this leads to a dependency of its electronic, optical, and thermal properties on the crystal orientation, hence enabling its use in anisotropic photoelectric and thermoelectric devices, similar to black phosphorus.^21,27,28^ In fact, Harrison and co-workers investigated this in-plane anisotropy through polarized Raman spectroscopy, establishing a clear correlation between the structural and optical in-plane anisotropies in exfoliated few-layer Ta_2_Ni_3_Te_5_.^21^

In this work, we systematically investigated the electronic, optical, excitonic, and vibrational properties of the Ta_2_Ni_3_Te_5_ monolayer, combining first-principles methods with a semiempirical approach for computational characterization. As indicated by the phonon dispersion spectrum, the dynamical stability was used to identify the monolayer’s active Raman and IR vibrational modes. Our results reveal an anisotropic behavior consistent with the analogous bulk structure. This anisotropy extends to the optical properties, where the optical response is highly sensitive to light polarization due to quantum confinement along the perpendicular basal direction.

## 2 Computational Details

The structural, electronic, and vibrational properties were obtained from simulations based on density functional theory (DFT)^29,30^ methods within the scope of the generalized gradient approximation (GGA)^31−35^ using the exchange-correlation (XC) functional proposed by Perdew–Burke–Ernzerhof (PBE).^31,36^ Because PBE underestimates the electronic band gap,^37,38^ causes self-interaction problems, and gives a poor description of weak interactions,^39−41^ we employed the hybrid XC functional proposed by Heyd–Scuseria–Ernzerhof (HSE06)^42,43^ to obtain a reasonable correction to the electronic band structure.

The Kohn–Sham (KS) equations were solved through the projector augmented wave (PAW) method,^44,45^ using the Vienna *Ab Initio* Simulation Package (VASP).^46,47^ For all simulations, a total energy convergence criterion of 10^–6^ eV was employed for the self-consistent cycle. To obtain the equilibrium structures, we optimize the stress tensor and minimize the interatomic forces with a plane-wave cutoff energy of 540 eV until the atomic forces on each atom were less than 0.01 eV/Å. We computed other properties with a lower cutoff energy of 304 eV.

For the integration of the Brillouin zone (BZ), we used a **k**-mesh of 2 × 11 × 1 for the electronic band structure and structural optimization calculations and a denser 4 × 22 × 1 **k**-mesh for the density of states (DOS) calculations. A vacuum distance of 21 Å was added along the *z*-direction to eliminate spurious interactions with the structure’s mirror images.

We used the Quantum Espresso (QE) package^48,49^ to obtain the phonon dispersion and the phonon density of states of the Ta_2_Ni_3_Te_5_ monolayer over the entire BZ, using truncated Coulomb interactions, -imposed by the assume_isolated flag,-;^50^ this method is beneficial to treat two-dimensional systems by avoiding the interaction with repeated images during the phonon calculation. The QERaman code,^51^ interfaced with QE, was then used to determine which of the 60 vibrational modes at the high-symmetry Γ point are Raman (R) active.

Determining the intensity of the R-active modes at several laser energy values can also be inferred, thus allowing a direct comparison with experimental data. These calculations were performed using a 60 Ry plane-wave energy cutoff, with a PBE exchange functional embedded in Troullier-Martins (TM) pseudopotentials (PPs).^52,53^ A 2 × 10 × 1 **k**-mesh and a small electronic temperature of 27 meV were added to smooth the numerical solutions and help reach convergence. It is important to note that the spin–orbit coupling (SOC) has been added self-consistently, only to point out the variation in the band structure. The phonon dispersion, excitonic effects, and Raman spectra were examined without including the SOC.

The maximally localized Wannier function Tight Binding (MLWF-TB) method was exploited to describe the electronic states and determine the MLWF-TB Hamiltonian using the Wannier90 code.^54^ The optical properties are then evaluated within the scope of the independent particle approximation (IPA) and through the solution of the Bethe–Salpeter equation (BSE)^55^ using the WanTiBEXOS package.^56^ It should be noted that the MLWF-TB Hamiltonian was obtained at an HSE06 level, directly from VASP, with a **k**-mesh of 6 × 11 × 1 considering p and d orbital projections for Ta and Ni, and s and p projections for Te. The BSE was solved using the Coulomb truncated 2D potential (V2DT)^57^ with a 7 × 32 × 1 **k**-mesh taking into account the lowest 12 conduction bands and the highest 12 valence bands; also a smearing value of 0.05 eV was applied for the dielectric function computation.

## 3 Geometry and Structural Properties

**Figure 1:**
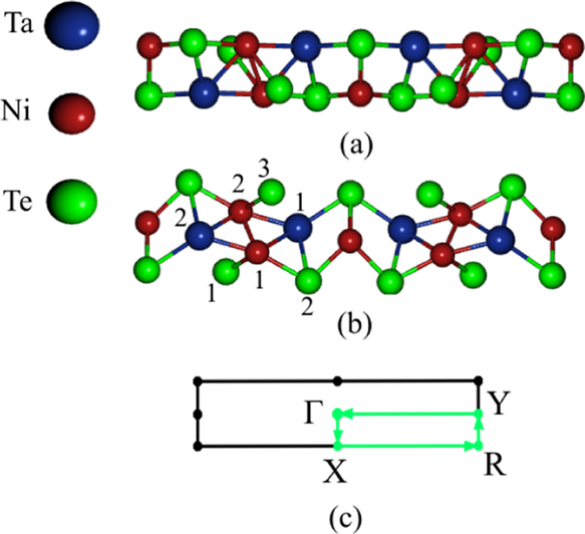
(a) Top view within the ***xy*** plane and (b) side view within the ***xz*** plane of a Ta_2_Ni_3_Te_5_ monolayer with blue, red, and green spheres representing Ta, Ni, and Te atoms, respectively. Some atoms are numbered to refer to different bond lengths. (c) The corresponding rectangular unit cell displaying the Γ (0,0,0), X(1/2,0,0), R(1/2,1/2,0), and Y(0,1/2,0) high-symmetry points.

The top and side views of the monolayer, as well as its two-dimensional (2D) Brillouin zone (BZ), are illustrated in Figure 1. The Ta_2_Ni_3_Te_5_ monolayer pertains to the *Pm* space group with equilibrium lattice constants *a* = 17.835 Å and *b* = 3.737 Å, consistent with the bulk experimental data.^21^ The structure has two Ni–Te bond lengths, with *d*_Ni_1_–Te_1__ = 2.58 Å and *d*_Ni_1_–Te_3__ = 2.80 Å. On the other hand, *d*_Ni_1_–Ta_1__ = 2.68 Å, *d*_Ni_1_–Ta_2__ = 2.62 Å, *d*_Ni_1_–Ni_2__ = 2.49 Å, and *d*_Ta_1_–Te_2__ = 2.89 Å. The cohesive energy per atom (*E*_coh/atom_), resulting from the arrangements of the different atoms in the ground state of the Ta_2_Ni_3_Te_5_ monolayer, is defined via:
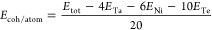


where *E*_tot_ is the total energy of the monolayer and *E*_Ta_, *E*_Te_, and *E*_Ni_ are the energies of isolated Ta, Te, and Ni atoms, respectively; the number “20” in the denominator refers to the total number of atoms in the unit cell. Our calculations predict an *E*_coh/atom_ of −4.492 eV, which, in principle, means that the structure is energetically favorable. We would like to add that the cohesive energy of the monolayer is lower than other 2D structures, such as silicene (−3.71 eV/atom),^58^ various phases of germanene (values between −3.60 and −3.39 eV/atom),^59^ and phosphorene (−3.61 eV/atom).^60^

## 4 Results and Discussion

### 4.1 Mechanical Properties

Studying the structure’s response to different strain types lets us obtain the mechanical properties of the Ta_2_Ni_3_Te_5_ monolayer. Since the unit cell is rectangular, a strain along the *x* and *y* directions will cause a change in the lattice parameters along these directions without actually varying the rectangular unit cell. As a consequence, the C_11_ and C_22_ elastic constants can be determined. A third elastic constant, C_12_, results from a biaxial strain within the *xy* plane that also preserves the rectangular unit cell.

**Figure 2:**
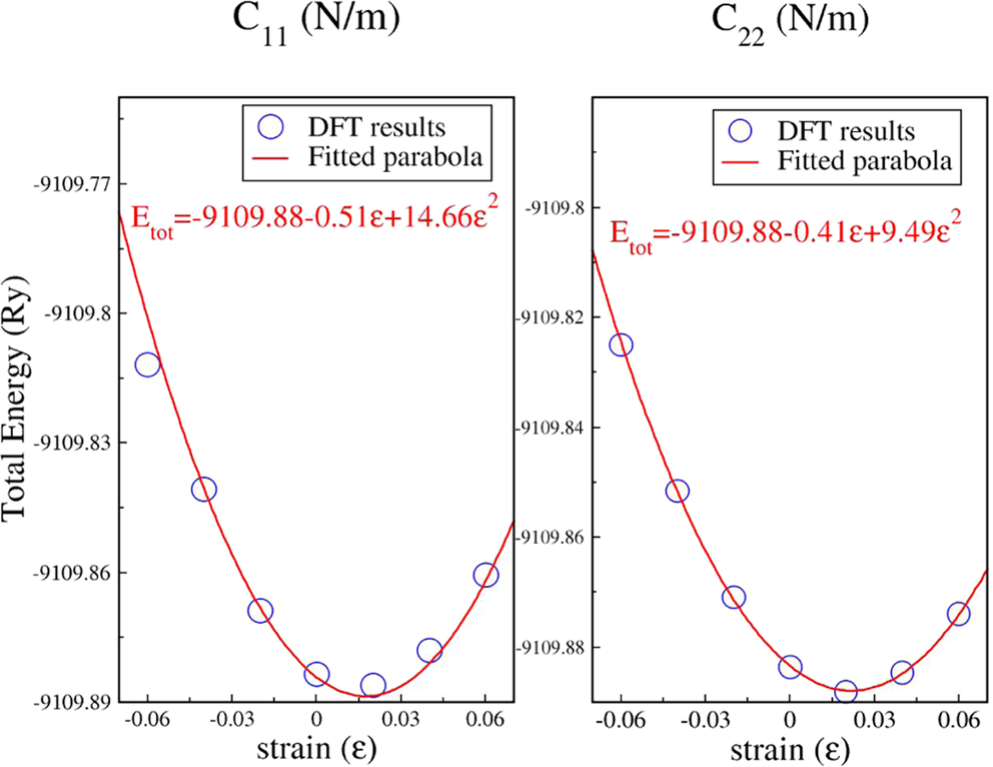
Elastic constants **C**_**11**_ and **C**_**22**_, obtained as a response to strain applied along the ***x*** and ***y*** directions, respectively.

Figure 2 exhibits the variation of the total energy (*E*_tot_) under compressive and tensile strain (ε) values, ranging from −6% to 6% and applied along the *x* and *y* directions. The DFT values are fitted to second-degree polynomials in ε, from which C_11_ and C_22_ were determined as , with *t* being the thickness of the Ta_2_Ni_3_Te_5_ monolayer and *V*_0_ the volume of the monolayer’s unit cell at equilibrium, equal to 1616.22 Å^3^. In this way, C_11_ and C_22_ will be vacuum-independent and in units of N/m.

From our calculations, C_11_ = 98.87 N/m, C_22_ = 64.05 N/m, and C_12_ = 21.56 N/m. Furthermore, the Poisson ratios ν_2D_^*x*^ = C_12_/C_11_ and ν_2*D*_^*y*^ = C_12_/C_22_ are 0.22 and 0.34, respectively. These values are vacuum-independent, exhibiting anisotropy along the *x* and *y* directions. They also illustrate the mechanical stability of the structure with C_11_ > 0, C_22_ > 0 and ν_2D_^*x*^ and ν_2D_^*y*^ < 0.5. A positive Poisson’s ratio confirms that the material is nonauxetic, contracting along the transverse direction when subjected to tensile forces. We would like to emphasize that the Ta_2_Ni_3_Te_5_ monolayer has passed the dynamical and mechanical tests of stability, which are consistent with its recent experimental synthesis.^61^

### 4.2 Electronic Band Structure and Density of States (DOS)

**Figure 3:**
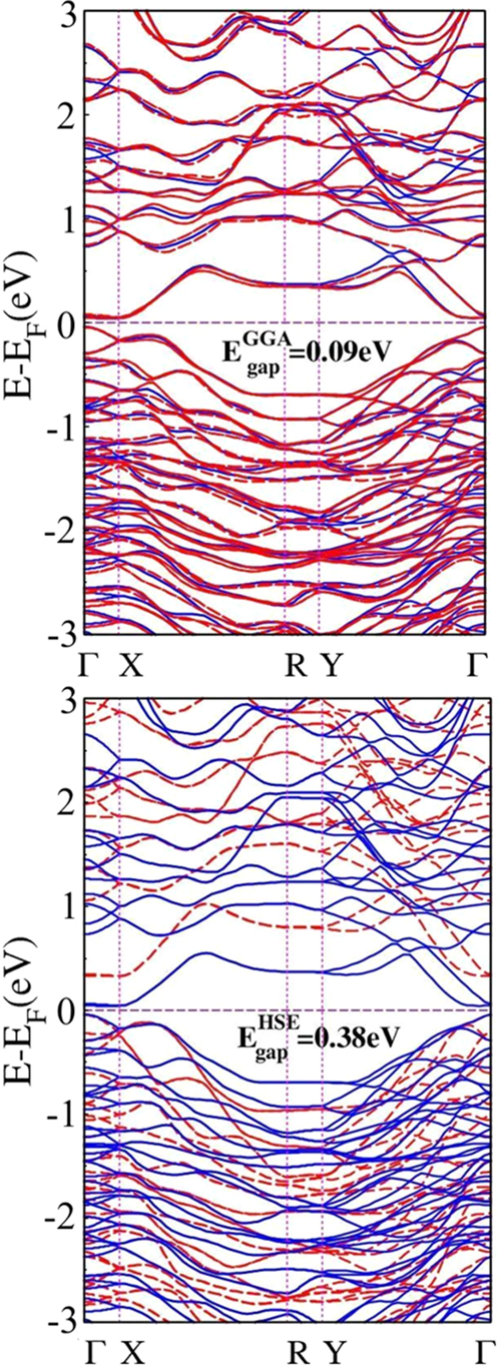
(Top) Electronic band structure with (in red) and without (in blue) SOC of the Ta_2_Ni_3_Te_5_ monolayer, with a band gap of 0.09 **eV**. (Bottom) Comparison between the GGA-PBE (blue) and the HSE06 (red) electronic band structures of the Ta_2_Ni_3_Te_5_ monolayer. The corrected band gap value is 0.38 eV. Here, SOC was not taken into account.

**Figure 4:**
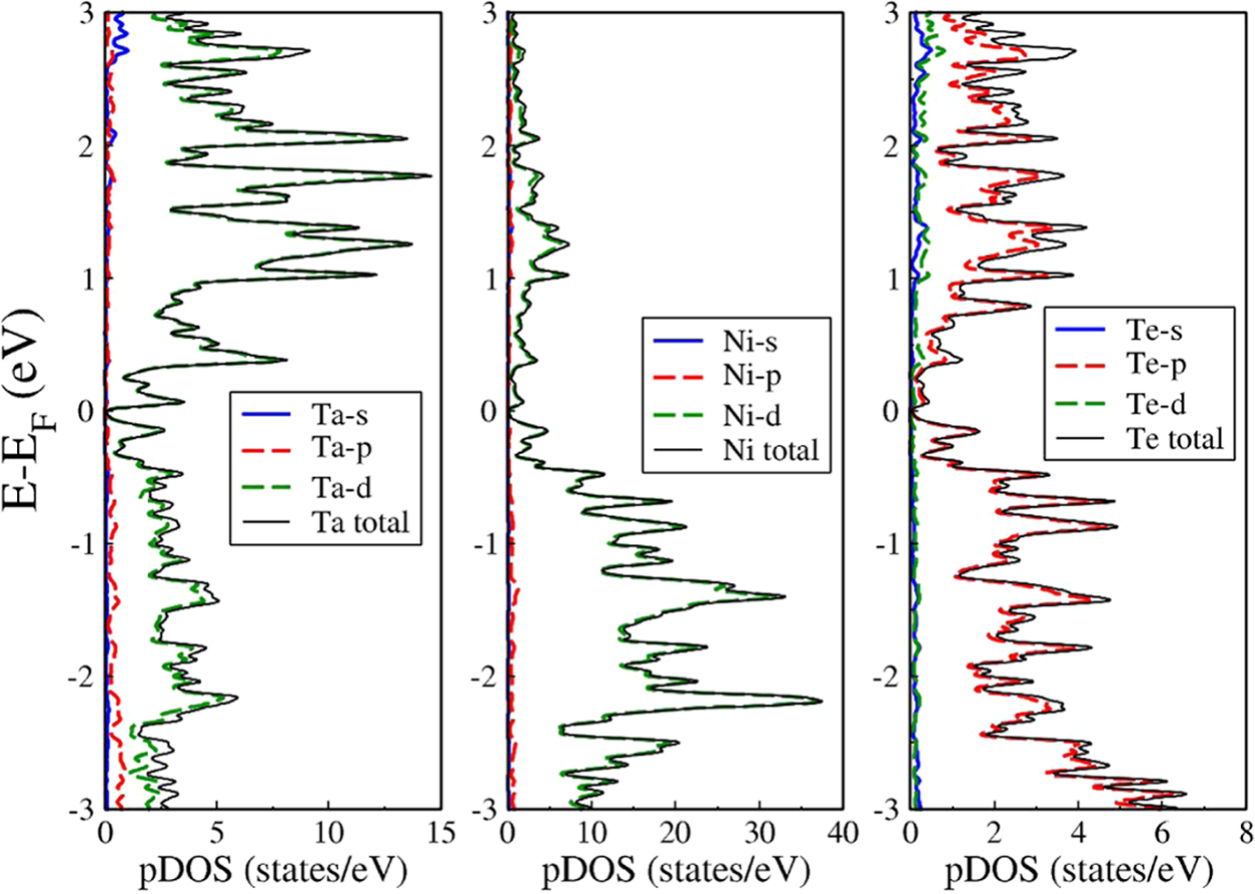
Partial density of states (pDOS) of the Ta (left), Ni (middle), and Te (right) atoms in the Ta_2_Ni_3_Te_5_ monolayer. The valence and conduction bands are mostly composed of the ***d*** orbitals of Ni and Ta and the ***p*** orbitals of Te. The Fermi level is set at 0 eV.

Our calculations show that the top of the valence band (VBM) occurs at the Γ point, while the bottom of the conduction band (CBM) is at a point along the Γ-X path. Figure 3 demonstrates that the Ta_2_Ni_3_Te_5_ monolayer is a semiconductor with an indirect small electronic band gap of 0.09 eV. This result is consistent with ref (22) (0.07 eV). The density of states (DOS) presented in Figure 4 shows that within the considered −3 to 3 eV energy range, both the valence and conduction bands are mainly contributed by the *d* orbitals of Ni and Ta, and the *p* orbitals of Te. More specifically, the most prominent DOS of the valence bands comes from the *d* orbitals of Ni; in contrast, the corresponding DOS of the conduction bands is primarily influenced by the *d* orbitals of the Ta atoms.

It is essential to mention that the bulk Ta_2_Ni_3_Te_5_ investigated in Jiang’s work^23^ confirms the semiconducting nature of this material with a small band gap of 31 meV using the projector augmented wave (PAW) method^44^ with a modified Becke-Johnson functional^62,63^ to describe the exchange potential. On the other hand, the 2D analogue has a band gap three (12) times larger on a PBE (HSE06) level. We note that the components of the magnetic moments were calculated for the PBE+SOC case, where we have obtained that *m*_*x*_ = −0.0005 μ_B_, *m*_*y*_ = −0.0005 μ_B_, and *m*_*z*_ = −0.0005 μ_B_ for the *x*, *y*, and *z* components, respectively. The values are negligible, thus discarding any significant magnetic behavior for the monolayer.

### 4.3 Phonon Dispersion and Raman and IR-Active Modes

Phonon calculations were carried out at the Γ high-symmetry point to obtain the modes of vibration of the monolayer. Since the unit cell contains 20 atoms, we expect 60 vibrational modes, the first three of which are acoustic (A) modes and the remaining 57 are optical (O) ones. At the Γ point, the modes decompose into:
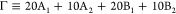
The three acoustic modes belong to the B_1_, B_2_, and A_1_ symmetries, and 10 out of the 20A_1_ modes are Raman (R) active.

**Figure 5:**
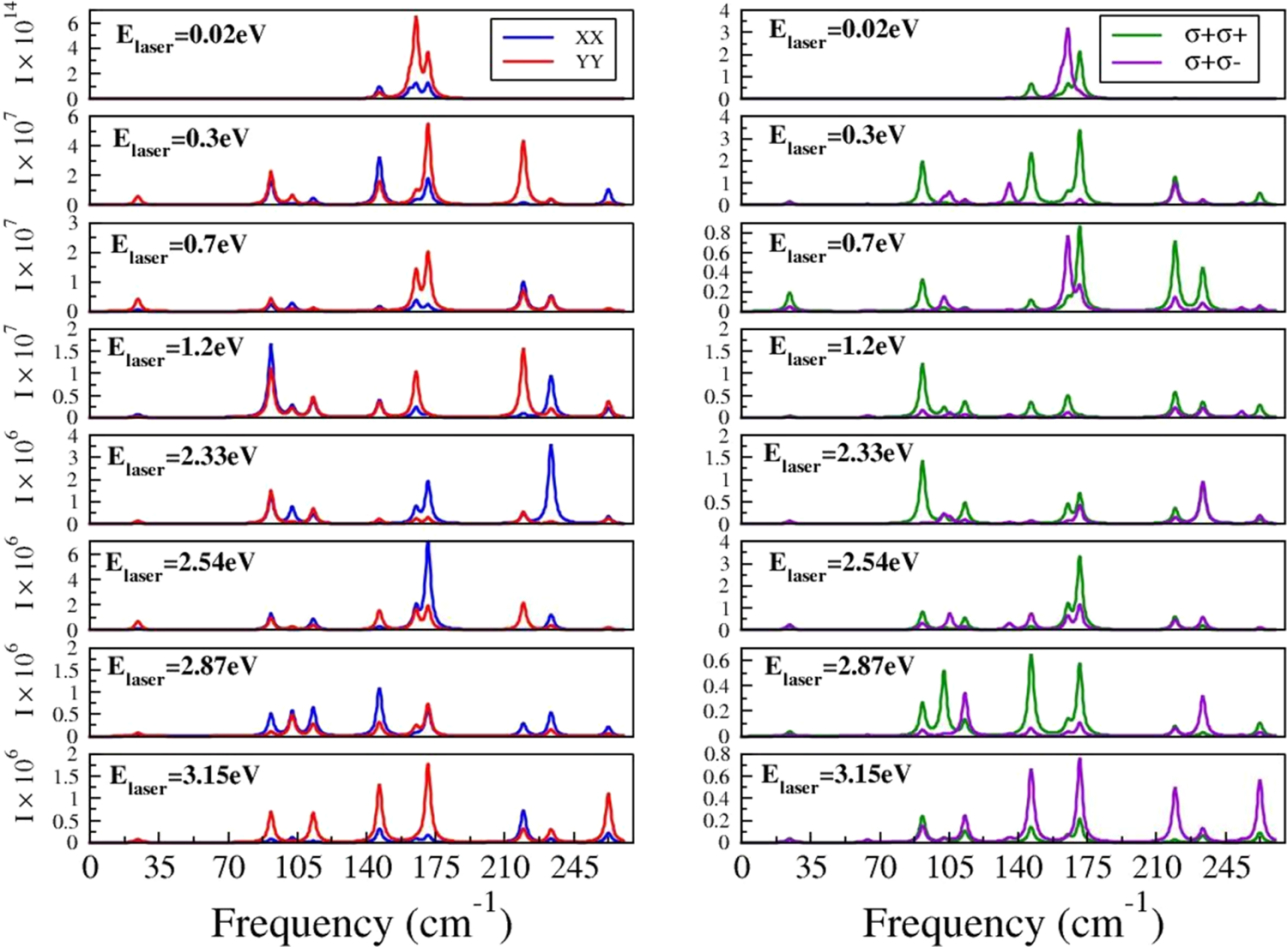
Raman spectra for a Ta_2_Ni_3_Te_5_ monolayer for different values of the laser energy (*E*_**laser**_). Results from the linearly (XX,YY) and circularly polarized light; both helicity-conserving (**σ + σ+**) and helicity-changing (**σ** + **σ**−) scattering cases are considered. “I” refers to the intensity of the observed peaks.

We estimated the Raman spectra for linearly and circularly polarized light to determine these modes using several laser energy values ranging from the infrared to the ultraviolet regimes. The results are presented in Figure 5. Our results are based on the assumption that the incident and scattered polarization vectors are parallel and that linearly polarized light propagates in a direction perpendicular to the plane of the Ta_2_Ni_3_Te_5_ monolayer. Only three peaks can be detected for laser energies (*E*_laser_) as low as 0.02 eV. As *E*_laser_ increases, other peaks emerge, varying in intensity. All in all, we can identify 10 such peaks. Focusing on *E*_laser_ = 2.33 eV, the peaks appear at 24.50, 91.73, 102.32, 113.28, 146.52, 165.15, 171.36, 219.59, 233.47, and 262.69 cm^–1^.

**Figure 6:**
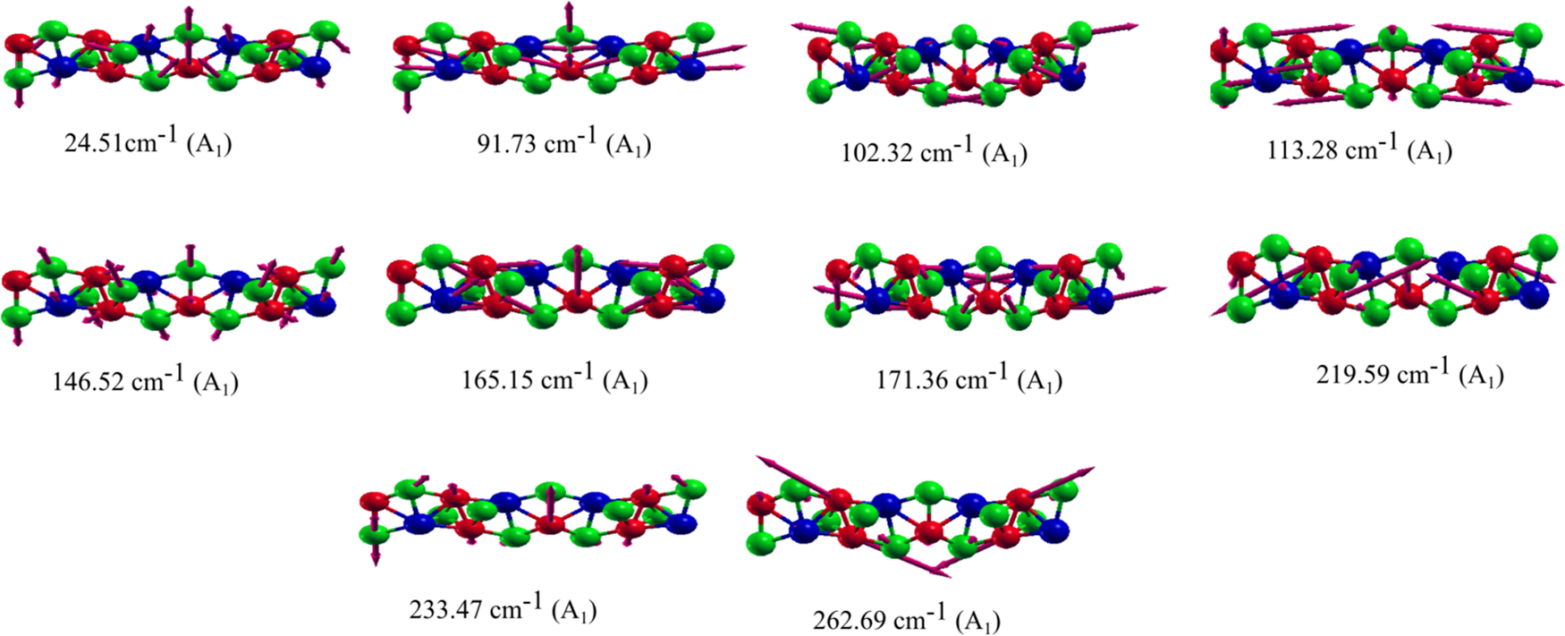
The 10 Raman-active modes showing the vibrations of the Ta, Ni, and Te atoms for each mode in the ***xz*** plane. The blue, red, and green spheres correspond to the Ta, Ni, and Te atoms, respectively.

**Figure 7:**
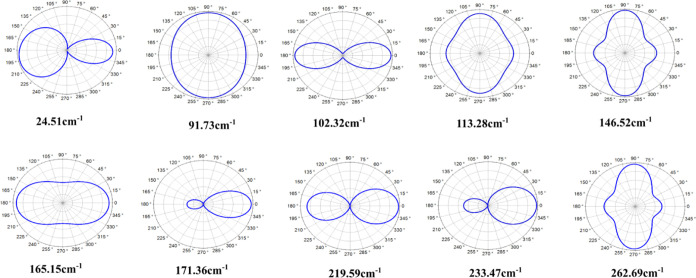
Based on complex Raman tensors, Raman intensities for circularly polarized light are displayed for the 10 active Raman modes of the Ta_2_Ni_3_Te_5_ monolayer.

The Raman-active modes are presented in Figure 6. We note that most of the vibrational modes do not exhibit displacement of the Ni atoms. On the other hand, mode 5 is mainly contributed by the vibration of the Te atoms. Figure 7 shows the Raman intensity for circularly polarized light for the 10 Raman-active modes. The intensity of mode 3 reaches its maximum for polarization angles θ = 0 or 180°, that is, along the ± *x*-directions. Modes 6, 7, 8, and 9 present maxima only at θ = 0°. In contrast, the intensity maxima of modes 2, 4, 5, and 10 occur when θ ∼ 90° and θ = 270°, that is, along the ±*y* directions, with mode 2 being quasi-isotropic. Mode 1 is an entirely distinct case where the maximum does not occur at 180° but rather at θ = 195.38°.

**Figure 8:**
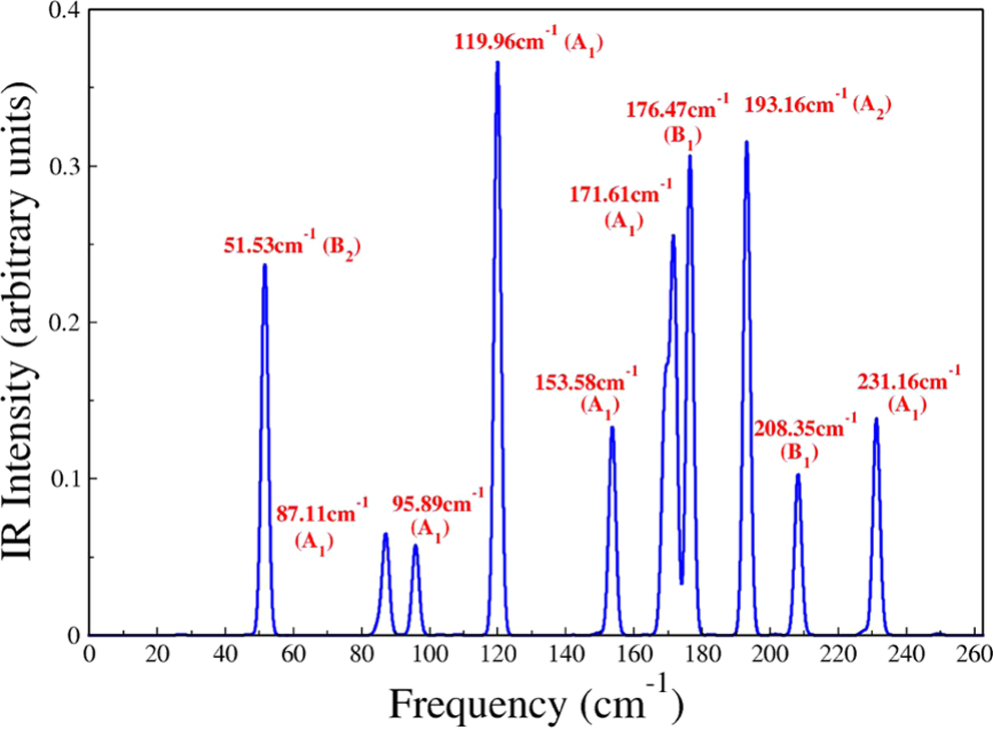
IR spectrum of the 2D-Ta_2_Ni_3_Te_5_ material. The frequencies and symmetry of the modes are also displayed.

We further calculated the IR intensities presented in Figure 8. The results indicate that 10 of the remaining modes are IR-active. More specifically, peaks of distinct intensities occur at 51.53, 87.11, 95.89, 119.96, 153.58, 171.61, 176.47, 193.16, 208.35, and 231.16 cm^–1^. Due to the closeness of the 171.61 and 231.16 cm^–1^ IR frequencies to those of the Raman-active ones (171.36 and 233.47 cm^–1^), we speculate that these modes can be both IR+R active.

**Figure 9:**
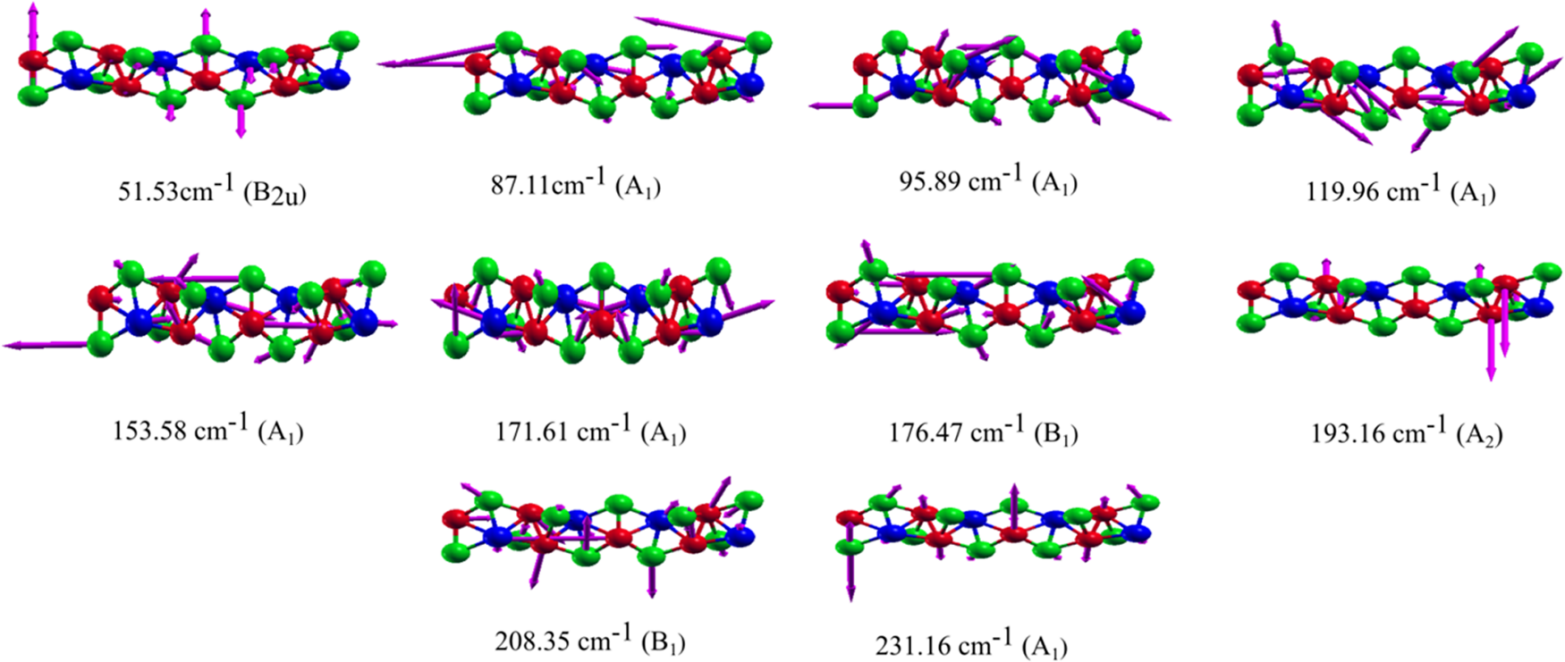
Identified 10 IR-active modes, corresponding to the IR spectrum peaks. The blue, red, and green spheres correspond to the Ta, Ni, and Te atoms, respectively. The vibrations are shown in the ***xz*** plane.

The atomic vibrations of the active modes in IR, shown in Figure 9, belong to symmetries A_1_, B_1_, and B_2u_. Modes 1 and 8 almost vibrate along the *z* direction and are composed mainly of vibrations of the Te and Ni atoms. The vibration of the Ta atoms is virtually absent in all IR modes.

In ref (21), the authors investigated the Raman-active modes of few-layer (FL) Ta_2_Ni_3_Te_5_ flakes, which were composed of two monolayers. To our knowledge, no work has yet discussed the monolayered structure. In total, 15 modes have been experimentally observed for the FL Ta_2_Ni_3_Te_5_ system emerging at 10, 28, 35, 63, 86, 92, 104, 116, 127, 138, 153, 166, 172, 204, and 228 cm^–1^. Some of these modes disappeared in the Raman spectrum of the monolayer, such as 10, 35, 63, and 86 cm^–1^, while others appeared, such as 262.69 cm^–1^.

**Figure 10:**
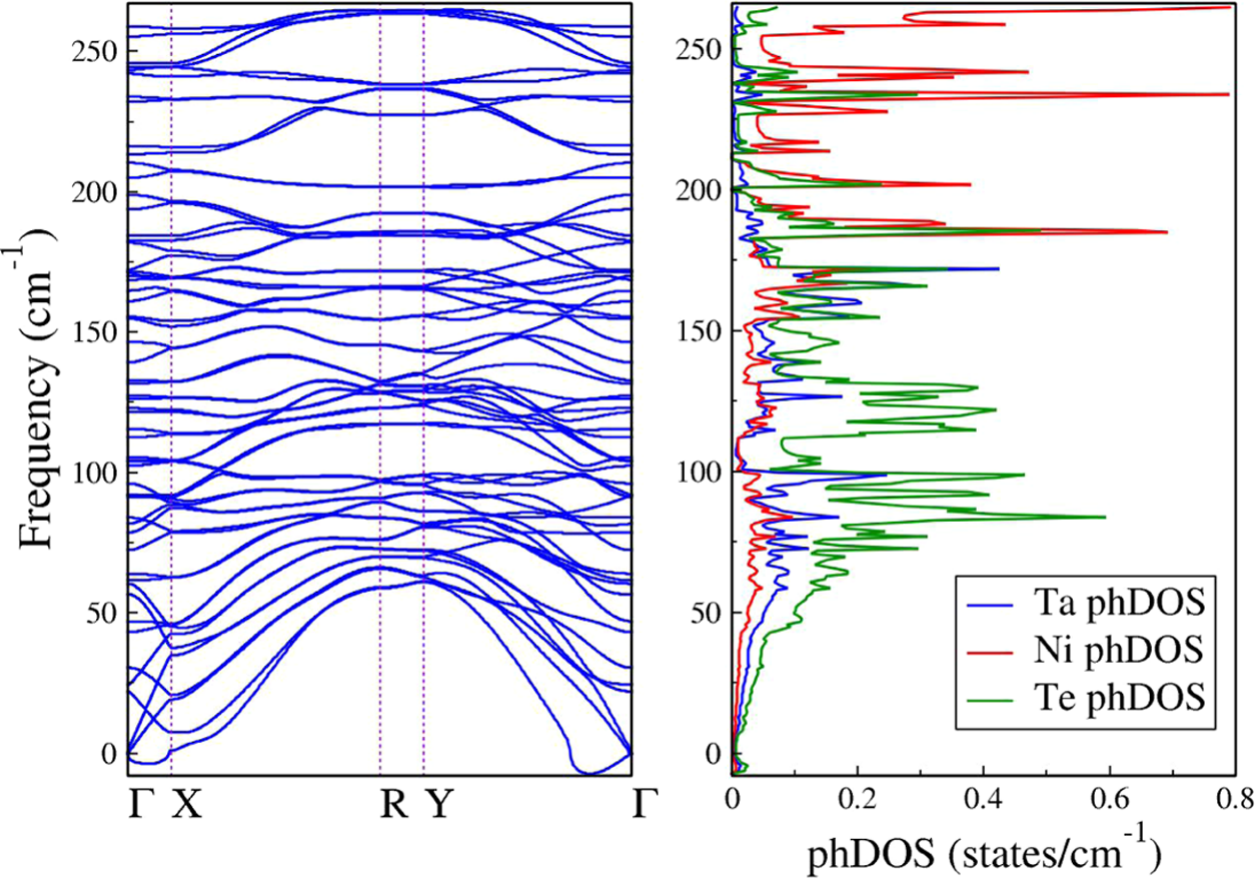
(Left) Phonon dispersion of the Ta_2_Ni_3_Te_5_ monolayer along the **Γ**-X-R-Y-**Γ** path. (Right) Phonon density of states and contribution of the Ta, Ni, and Te atoms to the vibrational modes.

To determine the overall contributions of the Ta, Ni, and Te atoms, the phonon density of states (phDOS) is plotted for the different phonon mode regimes, as demonstrated in Figure 10. The phononic band structure, also displayed in Figure 10, shows some slight negative frequencies in the flexural mode, not exceeding −7.6 cm^–1^, near the Γ point along the Y-Γ path. More minor negative frequencies, not ultrapassing −3.78 cm^–1^, extend along the Γ-X path. These frequencies do not indicate dynamic instability of the monolayer but instead represent numerical inaccuracies due to the diagonalization of the dynamical matrix. The phDOS exhibits the dominant contribution of the Te atoms up to ∼170 cm^–1^. For modes with higher frequencies, it is evident that the Ni atoms contribute the most.

### 4.4 Excitonic and Optical Properties

**Figure 11:**
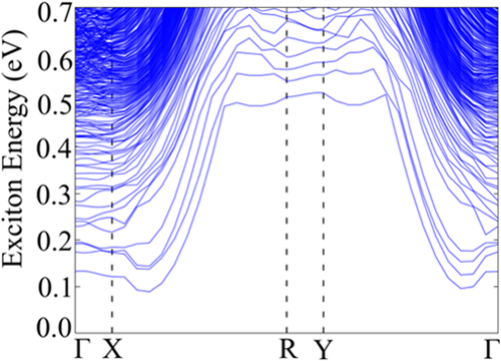
MLWF-TB HSE06 Ta_2_Ni_3_Te_5_ monolayer excitonic band structure.

From the excitonic band structure, shown in Figure 11, we can observe that the exciton ground state is indirect, with an exciton momentum between the *X* and *R* high symmetry points and an exciton binding energy of 287 meV which lies in the expected range for 2D materials.^9^ The direct exciton ground state, located at Γ, has a 0.13 eV gap and corresponds to the optical band gap. The presence of an indirect exciton ground state suggests the possibility of phonon-assisted optical transitions with excitation energies lower than the optical band gap; in fact, the difference between the exciton ground state and the optical band gap is very small, which makes it difficult to identify both peaks in the optical spectrum.

**Figure 12:**
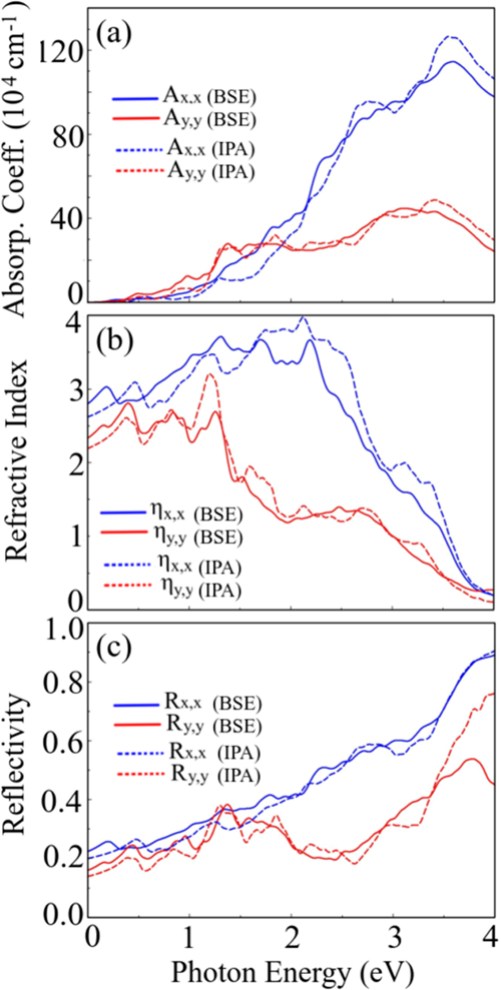
MLWF-TB HSE06 Ta_2_NiTe_5_ monolayer optical properties: (a) Absorption coefficient, (b) refractive index, and (c) reflectivity, all determined at the BSE (solid curves) and IPA (dashed curves) levels, considering linear ***x*^** (blue curves) and ***y*^** (red curves) polarizations.

The linear optical response of the Ta_2_Ni_3_Te_5_ monolayer is shown in Figure 12, with (BSE—solid curves) and without (IPA—dashed curves) excitonic effects for linearly polarized light along the *x̂* (blue curves) and *ŷ* directions (red curves). From the absorption spectrum, shown in Figure 12a, we can observe that for lower optical excitations in the infrared and at the beginning of the visible spectrum (around 1.5 eV), the optical response is very similar, independent of the light polarization. Conversely, we can see that for higher excitations, the system absorbs with a higher intensity for the *x̂* polarization case, showing a significant optical anisotropy in the visible and ultraviolet regions. The excitonic effects result in a slight red shift in the absorption spectrum, yet these quasi-particle effects do not considerably change the spectrum shape and intensities.

The refractive index and reflectivity are shown in Figure 12b,c, respectively. The refractive index is higher for polarization along the *x̂* direction. It registers the highest value in the visible region, decreasing as the photon energy moves to the ultraviolet region. Although the excitonic effects are evident, the changes are inconsiderable. Regarding the reflectivity, the opposite happens, as this factor is enhanced as the photon excitation energy increases. For photon energies higher than 2 eV, the optical anisotropy becomes more evident, showing a quasi-total reflectivity for photons closer to the ultraviolet region for light polarized along the *x̂* direction. For polarization along the *ŷ* direction and at BSE (IPA) level, the reflectivity at 4 eV is 40(80)%. The comparison between BSE and IPA optical responses is vital, as the excitonic effects are susceptible to the substrate where the monolayer could be placed. The higher the dielectric constant of the substrate is, the lower the excitonic effects.^9^

Besides, we have observed an interesting behavior when photon energy increases and is in the ultraviolet (UV) region; the absorption and refractive index tend to decrease while reflectivity increases, indicating that the material minimally absorbs ultraviolet radiation and has a high potential for reflection. This approach is particularly relevant for materials used to fabricate ultraviolet-blocking devices.

## 5 Conclusions

The Raman spectra, excitonic effect, and dynamic and mechanical stabilities of the novel Ta_2_Ni_3_Te_5_ monolayer have been investigated. The monolayer is an indirect small band gap semiconductor of 0.09 eV (0.38 eV) band gap value within the PBE (HSE06) approximation. It exhibits anisotropic properties, registering different mechanical responses to strain when applied along the *x* and *y* directions. It is also dynamically stable, except for minor negative frequencies near the Γ point in the corresponding phonon dispersion spectrum, primarily due to numerical inaccuracies in the dynamic matrix diagonalization process. Its phonon DoS illustrates the dominant contributions of the Te and Ni atoms to the vibrational modes of the monolayer.

The Raman spectrum exhibits an increase in the number of peaks upon an increase in the laser energy values. We have identified 10 peaks corresponding to this monolayer’s 10 Raman-active modes for a typical laser energy of 2.33 eV. We have further determined 10 infrared modes characterized by the predominant vibrations of the Te and Ni atoms.

The excitonic band structure for this monolayer possesses a binding energy within the expected range for 2D materials and an indirect exciton ground state, which is a signature of the possibility of the occurrence of phonon-assisted optical transitions with excitation energies smaller than the optical band gap. Additionally, the system is highly anisotropic, causing an optical response dependent on incident light polarization. Moreover, the system shows a higher reflectivity for light polarized along the *x̂* direction in the visible and ultraviolet regions, making the Ta_2_Ni_3_Te_5_ monolayer appropriate as a polarizing filter.
